# Protecting Children from Toxic Waste: Data-Usability Evaluation Can Deter Flawed Cleanup

**DOI:** 10.3390/ijerph17020424

**Published:** 2020-01-08

**Authors:** Kristin Shrader-Frechette, Andrew M. Biondo

**Affiliations:** 1 Department of Biological Sciences, 100 Malloy Hall, University of Notre Dame, Notre Dame, IN 46556, USA; 2 Department of Economics, 3060 Jenkins Nanovic Hall, University of Notre Dame, Notre Dame, IN 46556, USA; abiondo@nd.edu

**Keywords:** children, data-usability evaluation (DUE), environmental justice, Ninyo and Moore, Pasadena, California, perchloroethylene (PCE), remediation, toxic waste, Trammell Crow, trichloroethylene (TCE)

## Abstract

Nearly 25 percent of US children live within 2 km of toxic-waste sites, most of which are in urban areas. They face higher rates of cancer than adults, partly because the dominant contaminants at most US hazardous-waste sites include genotoxic carcinogens, like trichloroethylene, that are much more harmful to children. The *purpose* of this article is to help protect the public, especially children, from these threats and to improve toxics-remediation by beginning to test our *hypothesi*s: If site-remediation assessments fail data-usability evaluation (DUE), they likely compromise later cleanups and public health, especially children’s health. To begin hypothesis-testing, we perform a focused DUE for an unremediated, Pasadena, California toxic site. Our DUE methods are (a) comparing project-specific, remediation-assessment data with the remediation-assessment conceptual site model (CSM), in order to identify data gaps, and (b) using data-gap directionality to assess possible determinate bias (whether reported toxics risks are lower/higher than true values). Our *results* reveal (1) major CSM data gaps, particularly regarding Pasadena-toxic-site risks to children; (2) determinate bias, namely, risk underestimation; thus (3) likely inadequate remediation. Our discussion shows that if these results are generalizable, requiring routine, independent, DUEs might deter flawed toxic-site assessment/cleanup and resulting health threats, especially to children.

## 1. Introduction

By the year 2035, the US Environmental Protection Agency (EPA) projects (in its latest all-inclusive report) that 350,000 of the nation’s toxic-waste sites will need remediation, including military, other federal, state, and private sites [1]. Most are in densely populated urban locations whose residents include disproportionate numbers of minorities and people living below the poverty line. Although these near-toxic-site residents must confront statistically significant increases in cancer and other diseases [2], children face the worst threats [3,4,5,6]. Roughly 25 percent of all US children live within 2 km of a toxic-waste site [7] (p. 159). Just at one of thousands of such US sites, in Portland, Oregon, up to 25,000 people have been potentially affected by carcinogens that have no safe dose. Their death rates from bile-duct, kidney, pancreatic, and stomach cancer are 200 percent to 800 percent higher, depending on the cancer, than those of the unexposed population [8].

Across the US, tens of millions of people also are at risk because of contaminated drinking water. In more than half the nation’s public-drinking-water systems, levels of the genotoxic carcinogen trichloroethylene (TCE) exceed what is safe for children’s health [5,9].

*Why are children more vulnerable to such contaminants?* Children and pregnant women are more vulnerable than adults to toxic wastes because fetuses and children have special windows of increased vulnerability during different developmental periods. Exposures during these critical windows could cause permanent structural, organ-system, or functional deficits, such as TCE-induced heart defects from maternal, first-trimester, pregnancy exposure. Fetuses and children also experience rapid growth and development, thus a higher rate of cell division and differentiation, and have less developed immunological and detoxification systems, and more efficient mechanisms for blood-brain transport of contaminants—all of which may increase their susceptibility. Children likewise receive greater toxic exposures, per kilogram of body weight than adults, because they take in proportionately more food, water, and air. In addition, younger children have greater (than adult) pollutant exposures because of lactation, hand-to-mouth activity, and a breathing zone closer to soil contaminants. Because children have a longer lifetime to express pollutant damage, they also experience greater harm than adults from contaminants [5] (pp. 15, 167–169, 202, 258–262).

Because of children’s increased susceptibility to genotoxic carcinogens like TCE, US EPA recommends using age-dependent adjustment factors (ADAFs) to calculate children’s heightened risks. The ADAF risk multiplier is 10 for children < 2 years of age, 3 for children 2 to <16 years of age, and 1 for children ≥ 16 years of age [5] (p. 183).

*What can be done to protect the thousands of victims of toxic sites?* To begin to answer this question, the authors (1) document the flawed science, fraudulent cleanups, and underestimates of pollutant-risk that appear to have harmed thousands of toxic-site victims. They also (2) conduct preliminary testing of one hypothesis about how to help protect potential toxic-site victims by performing a focused data-usability evaluation (DUE) of remediation documents for a prominent US toxic site. (A DUE assesses how missing, questionable, or rejected data affect the data’s usability for the project; it evaluates whether the provided data meet the data needs that are specified in the project’s conceptual site model (CSM).) The authors’ hypothesis is that if routine, independent, pre-cleanup, remediation assessments/plans for perchloroethylene (PCE)- and TCE-contaminated sites fail focused (limited-scope) data-usability evaluations (DUEs), they are more likely to compromise later cleanup and public health, especially child health.

The authors’ hypothesis focuses on PCE/TCE contamination because most US toxic sites contain these chlorinated industrial solvents, and they are carcinogens, neurotoxins, and developmental toxins. Although PCE/TCE have been extraordinarily useful as cleaners, degreasers, and solvents, as well as ingredients in adhesives, clothing, foodstuffs, pesticides, pharmaceuticals, and textiles [5,6], either PCE, TCE, or their breakdown products (such as vinyl chloride and dichloroethylene) are genotoxic carcinogens, have no safe dose, and are much more harmful to children than adults [5,6]. US regulators rank them in the top four-most-harmful toxins that pose “the most significant potential threat to human health” [5,10]. US regulators also rank them in the top four-highest-exposure toxins “to which the public is being exposed” [11].

*Why are some of the deadliest waste-site toxins, PCE and TCE, precisely those to which the public—and especially young children—are most exposed?* One reason is inadequate attention to nearly a century of PCE/TCE health warnings. By 1920, PCE and TCE were high-production-volume chemicals [12] that had already killed both farm animals [13] (p. 119) and experimental animals used in very low-dose studies of PCE as an antihelminthic [14]. Although the 1930s medical literature, including *The New England Journal of Medicine*, warned against PCE/TCE medicinal use, workplace exposure, and industrial poisoning, use of PCE and TCE for anesthetics and medications continued through the 1970s. By 1945 states, like California, had adopted PCE regulations [12,14], and US National Cancer Institute experiments had shown that chlorinated solvents can cause liver cancer in animals [13] (pp. 119–120). Shortly after, UK scientists warned that TCE groundwater pollution already was a persistent problem, that even tiny concentrations were toxic [13,15,16].

By 1948, California’s Public Health Director cautioned that, without better groundwater regulations, chlorinated solvents like PCE and TCE would cause “a backlog of water pollution over the State…a plague comparable to the air pollution in Los Angeles” [13] (p. 107). By the early 1950s, sensitive analytic methods had made PCE/TCE groundwater regulations feasible. Yet, because US regulators faced pressure from industrial-solvent producers/users, the US did not propose PCE/TCE drinking-water regulations until 2011—more than 50 years later [5,13]. The same apparent pressures continue today [13,14,17]), halting US EPA’s planned, 2016 ban on many TCE uses [9,18]. Nevertheless, safety and liability concerns have caused declines in US PCE/TCE production that, 40 years ago, was 340 million and 272 million kilograms/year, respectively [12,19].

These declines began partly because the 1971 deaths of three young children drew widespread international attention to PCE/TCE health and regulatory concerns. The children were the first in a score of child leukemias and deaths, all in one neighborhood of Woburn, Massachusetts [20,21,22]. Soon other apparent PCE/TCE-caused child cancers appeared, e.g., [23,24,25,26,27]. Because PCE and TCE are volatile organic compounds (VOCs), their groundwater contamination and carcinogenic-soil vapors/gases can travel underground for scores of meters—then migrate laterally and vertically, contaminating drinking water and causing carcinogenic “vapor intrusion” into homes/buildings located above or near the subsurface toxins. PCE/TCE contamination thus has claimed thousands of victims, especially children, in areas such as St. Louis Park, Minnesota [28]; Rockton, Illinois [29]; Lise, Illinois [30]; Franklin, Indiana [31,32,33]; Riverside, Ohio [34]; Dayton, Ohio [35]; Cape Cod, Massachusetts [26,36]; Dracutt, Massachusetts [37,38]; Endicott, NY [39]; N. New Jersey [26]; Pompton Lakes, NJ [9,40]; Dover, Delaware [41]; Wake Forest, North Carolina [42,43,44]; Camp LeJeune, North Carolina [26,41,45,46]; Jacksonville, Florida [41]; Fort McClellan, Alabama [41,47,48]; Oklahoma City [41,49] Scottsdale, Arizona [50]; Tucson, Arizona [26]; Yuma, Arizona [41]; Los Angeles [51,52]; China Lake, California [53]; Mountain View, California [54]; San Francisco, California [53,55,56,57]; Portland Oregon [15,58]; Whidbey Island, Washington [59]; Mountain Home, Idaho [41]; and Denver, Colorado [26].

*Why does PCE/TCE harm continue, despite nearly a century of health warnings?* One reason is that there are no inexpensive, timely, and effective PCE/TCE-remediation technologies [60,61]. Instead, cleanup requires at least year-long groundwater/soil/soil-vapor sampling, using multiple lines of evidence to avoid “false negatives” that arise from fluctuations in variables such as temperature, barometric pressure, soil moisture, soil type, and wind [62,63]. Remediation is difficult because PCE/TCE can form dense, non-aqueous phase liquids (DNAPLs), heavier-than-water contaminants that sink very deeply into soil and groundwater. Scores of meters deep, these DNAPLs produce carcinogenic vapors that intrude into surface buildings [63]. Yet because their depth makes removal difficult and costly, DNAPLs often cause long-term contamination of soil, groundwater, and surface buildings [64,65]. Given the economic incentives for inadequate PCE/TCE-assessment/remediation, there is a need to test our hypothesis that DUEs might help deter flawed cleanups.

**Figure 1:**
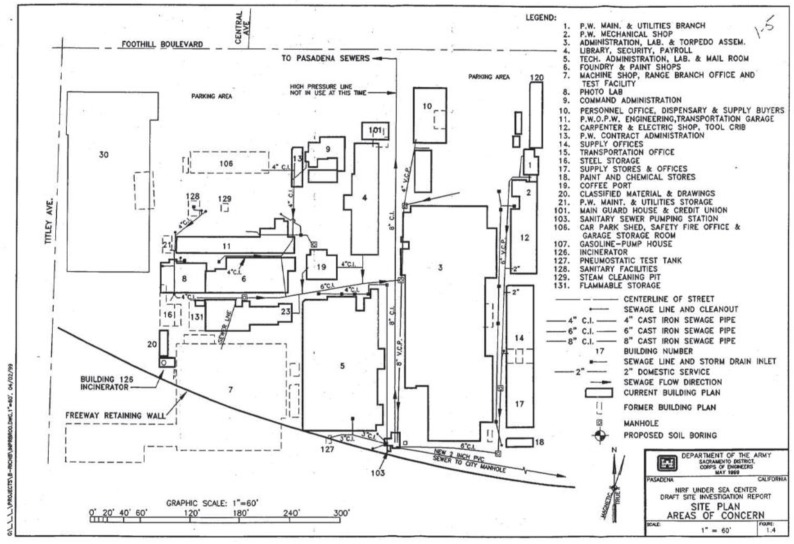
Map of the former US Naval Ordnance Test Station, Pasadena. (Map is from US Army Corps of Engineers, Draft Site Investigation Report, NIRF Under Sea Center Site Inspection, Figure 1.4).

*To begin to test this hypothesis*, the article conducts a focused DUE for a Pasadena, California, US Navy toxic site, the US Naval Ordnance Test Station (see the authors’ Figure 1). It is listed as the Naval Information Research Foundation, Envirostor ID 19970020, on the DTSC list of hazardous- waste sites <https://www.envirostor.dtsc.ca.gov/public/profile_report.asp?global_id=19970020>.

Although the Pasadena site is not on the Superfund list, its cleanup must follow Superfund remediation rules, partly because its carcinogenic industrial solvents are up to 744,000 times above allowed levels (see the authors’ Table 1). From World War II through the mid-1970s, the site was used for secret, classified development/testing/manufacturing of weapons, especially Polaris (nuclear) missiles, torpedoes, anti-submarine/other classified weapons, and fire-control systems [66,67,68]. The toxic site’s main “risk drivers” are PCE and TCE, followed by carbon tetrachloride (CT) and dibromochloromethane. Other site contaminants include lead, mercury, cadmium, hexavalent chromium (from the onsite foundry and weapons-fabrication shops); dioxins and furans from the site’s 5 former incinerators; Total Petroleum Hydrocarbons (TPH) from diesel/motor oil/gasoline/solvents in underground-storage tanks; semi-volatile organic compounds such polycyclic aromatic hydrocarbons (PAHs), especially benz(a)anthracene, benzo(a)pyrene, benzo(b)fluoranthene, and dibenz(a,h)anthracene; PCBs; radioactive materials; weapons’ propellants such as perchlorate; and fire-fighting contaminants such as polyfluoroalkyl substances (PFAS) [68] (pp. 12–14).

Since the Navy left the site 45 years ago, prospective purchasers hired consultants to assess site toxicity/needed remediation. After each assessment, each deal fell through [66]. Hence the site has never been cleaned up—only walled and paved over [68] (pp. 8, 134). However, in 2007 Ninyo and Moore did risk/remediation studies for the largest US commercial developer, Trammell Crow [69]. It now hopes to purchase the site and build shops and 550 small apartments on it [68] (pp. 1–2, 41).

Although thousands of people will live on the redeveloped toxic site, the developer will do no preconstruction groundwater testing; remove only 11 small, localized, metals-hotspots [70] (p. 8), [67] (pp. 20, 29); and leave most carcinogens, including TCE/PCE, “in place” onsite [70] (pp. 4, 8). After residents, including children, move into the 550 apartments, the developer says he can test indoor air, then do any needed PCE/TCE cleanup at that time. However, he admits that this later cleanup could cause “more than a year” of higher cancer rates for site residents. [67] (pp. 35–43).

Will the developer’s minimal toxic-site cleanup and reliance on land-use controls protect public health [72]? One way to begin to answer this question is to test the authors’ hypothesis.

*The research findings of this hypothesis-testing article are significant* for at least 5 reasons. (1) They may help prevent PCE/TCE health harm, like that suffered by 60 Indiana youngsters who have been diagnosed with, or died from, rare TCE/PCE-associated blood/brain cancers from a local toxic site [31,73,74]. (2) As US EPA has recommended since the 1980s [74], the findings also may provide an economical, noninvasive, early-warning method, a focused DUE, to help avoid wasting money on ineffective toxics’ cleanups. (3) The findings likewise may help deter scientific fraud in site remediation [75,76,77,78,79,80,81], like the fraud that has just occurred in San Francisco’s Hunter’s Point [55,56,57,82,83]. (4) They also may promote more timely remediation of the world’s largest cleanup, the US legacy from the Cold War [75]. (5) Given difficulties policing cleanup contractors, the findings likewise may help reduce legal/financial liability for toxic-waste sites [80] (p. 625), [81].

## 2. Materials and Methods

Recall that a DUE assesses whether the provided data meet the data needs specified in the project’s CSM. To begin preliminary testing of the authors’ hypothesis, the article conducts a focused or limited-scope DUE of *two sets of remediation-assessment materials*, both authored by Ninyo and Moore for developer Trammell Crow. The first set of materials is the 500-page Remedial Investigation. It argues for redeveloping the toxic site before doing any groundwater testing or removal of the site’s main risk drivers, carcinogenic VOCs; instead it argues for removing only 11 small, localized metals-hotspots [67,70]. It claims that although site TCE, PCE, and other VOCs are nearly a million times higher than allowed (see the authors’ Table 1), they present an average, indoor-air, lifetime risk of only about 1 cancer in every 3000 persons exposed [67]. (However, the study’s source data show that the outdoor/ambient-air, lifetime risk is as high as 1 cancer in every person exposed [67,84] (see the authors’ Table 2), an apparent DUE problem [85]). The Remedial Investigation also says that if carcinogenic vapors enter future apartments, they can be remediated later [67] (p. 34), [86] (p. 4651).

The second set of site-assessment materials is the Removal Action Workplan, also roughly 500 pages. It evaluates 3 post-construction, site-remediation approaches. Alternative 1 is no action. Alternative 2 is preconstruction removal of only 11 localized metals-hotspots then, if needed, post-construction remediation of PCE, TCE, and other carcinogenic solvents—which the developer admits could cause “more than a year” of high cancer risks to residents while carcinogens are being reduced [67] (pp. 35–43), [86] (p. 50). Alternative 3 is like alternative 2, but it includes remediation of PCE, TCE and other carcinogens during site construction. Although the developer admits alternative 3 “would eliminate any potential” threats to health, he plans to use the riskier alternative 2, as he says alternative 3 “would be a costly and time-intensive process” [86] (pp. 47, 51). [81].

Note that *no site-remediation materials include* either *a data-usability evaluation (DUE*) or a current Data Quality Analysis (DQA). In 2006, an earlier prospective site purchaser performed a brief DQA of his partial soil-sample set [67] (pp. 2–3). However, site documents include no current DQA of 2007–2019 data, and no one has ever conducted a DUE. DQA is analogous to data verification, evaluating the analytical quality of data in themselves, whereas DUE is analogous to data validation, assessing data quality, in light of project needs [87] (p. 3). DQA thus assesses the precision, accuracy, representativeness, comparability, completeness, and sensitivity of a specific data set, whereas DUE assesses the adequacy of all data sets for a specific project, like site remediation [74,85] (pp. 1, 10). DUE especially evaluates “how missing, questionable, or rejected data affect the data’s usability” [88] (pp. 9, 48, 42)—and whether any bias “affects the usability of the data for the intended purpose” [85] (p. 18). Determinate bias is directional, reporting risks that, overall, are lower/higher than true values. Indeterminate bias is not directional but shows “conflicting bias” or “poor analytical precision,” so that because some reported risks are lower, and some higher, than true values, the over- and under-estimates balance out, in terms of the overall risk-data directionality [85] (pp. 17, 35–43).

Regarding methods, the article conducts a “focused” or limited-scope DUE [88] (p. 41). That is, it limits itself to the DUE sub-methods most critical for detecting determinate and false- negative bias that could cause risk underestimates and human-health harm. The first DUE sub-method is (1) comparing Pasadena remediation-investigation data with the project’s conceptual site model (CSM) [89], to identify any potential data gaps [85] (pp. 6, 40), [74] (p. 23). The second DUE sub-method is (2) determining the directionality of any data gaps, so as to determine potential determinate bias (and risk underestimation) because of such preceding gaps [85] (p. 18).

The *first sub-method of the DUE*, data-gap identification, examines the 6 main risk factors of the CSM: site contaminants, sources of environmental releases, toxin-transport pathways, exposure pathways, exposure areas, and receptors (potential risk victims) [89]; [74] (p. 18); [85] (pp. 6, 40), for all of which toxic-site, risk/remediation plans are supposed to provide risk/toxicity data. Because any data gaps in these 6 areas could compromise later cleanup, the goal of this first sub-method is to assess whether the data-sampling tables (in site-remediation documents) cover all aspects of the CSM, needed for remediation [89]; [74] (p. 23); [85] (pp. 6, 40). The four main steps in this first sub-method are (1) identifying data needs in the 6 CSM risk factors; (2) locating all data sets for these 6 risk factors such as sources, pathways, etc.; (3) comparing data needs in (1) with reported data in (2) to discover possible gaps; and (4) assessing the potential for each data gap, if any, to positively or negatively affect later remediation and human health [74,85].

The *second DUE sub-method*, determining directionality of any data gaps, so as to assess any determinate bias, includes 3 main steps. These are (1) identifying each data gap, if any; (2) using site data to determine whether reported risk values are lower/higher than they likely are and whether assessors ignored any above-allowed-level risks; and (3) assessing each data gap for its contribution to risk under/over-estimates, thus to directionality and determinate bias [74,85].

## 3. Results

To assess potential CSM data gaps, the first DUE method followed the four main steps, outlined above. Subsequent sections use these steps to survey the three apparent CSM remediation-data gaps. These are failure to calculate *groundwater risks*, *airborne risks* from carcinogenic vapors/particulate matter (PM) and most *risks to receptors/victims*, especially site-resident and area-resident children.

### 3.1. Data Needs in the Conceptual Site Model (CSM)

The CSM reveals 4 sets of data-assessment needs. These are soil-contaminant-transport mechanisms, exposure media, exposure routes, and potential receptors. CSM soil-transport data are needed for mechanisms of (1) direct contact, (2) airborne PM, (3) volatilization to vapor, (4) storm-water runoff, and (5) migration to groundwater. CSM exposure-media data are needed for (6) soil, (7) air, and (8) groundwater. CSM data for contaminant-exposure routes are needed for soil (9) ingestion, (10) inhalation, and (11) dermal contact; (12) air inhalation; and groundwater (13) ingestion, (14) inhalation, (15) dermal contact. Finally, CSM data are needed for potential receptors (16) commercial/construction workers and site residents, and (17) area residents [89].

### 3.2. Three Apparent Conceptual Site Model (CSM) Data Gaps in Site Risk/Remediation Documents

Given the 17 CSM data needs, site-remediation assessments appear to fully cover 5 of 17 needs. That is, no obvious data gaps appear in remediation-investigation data for (1) direct-contact exposure to (6) soil through routes of (9) ingestion, (10) inhalation, and (11) dermal contact.

However, the *first* and likely greatest data gap is no coverage of 6 CSM areas of groundwater risk. These are exposures through (4) storm-water runoff and (5) contaminant migration to (8) groundwater, via routes of (13) ingestion, (14) inhalation, and (15) dermal contact by receptors. A *second* and partial data gap is incomplete coverage of 4 CSM-data needs. These are exposure to (2) PM and (3) contaminant soil vapor in (7) air, through the route of (12) inhalation. A third and partial data gap is incomplete coverage of two CSM-data needs, risks to receptors/victims, (16) site workers and residents, and (17) area residents [89]. Should these data gaps be closed?

### 3.3. The First, or Groundwater Exposure-Route, Data Gap

As already noted, although the CSM shows (8) groundwater is a potential exposure medium, there are no site groundwater-risk data/samples. Because remediation documents claim groundwater risks are “unknown” [86] (p. 36), they are not included in the site Human Health Screening Evaluation [67] (see the authors’ Table 3). Subsequent sections show that assessors/regulators have offered five attempted scientific justifications for the groundwater-data gap—all of which appear to fail.

#### 3.3.1. Justifying CSM Groundwater-Data Gap (8) by Ignoring Groundwater Evidence

Project regulators Nicholas Ta and Patrick Hsieh of California DTSC defend the groundwater- data gap, saying “There is no data indicating groundwater at the site is contaminated or that the site is the source of groundwater contamination” [90] (p. iii). Yet this claim is doubtful for 5 reasons.

*First*, DTSC itself said site-groundwater contamination is likely and criticized failure of site documents to test groundwater. DTSC’s official evaluations of site documents say groundwater is likely a VOC “source” and should be tested, preconstruction, given known contaminant migration, e.g., VOCs at the deepest levels tested, 46 m [67] (pp. 20, 39); [70] (pp. 1, 8–10); [86] (pp. 31, 36, 44).

*Second*, state data also indicate site-groundwater contamination—as the closest state-ground water-monitoring well (well 1), less than 2 km down-gradient from the site, is contaminated with the site’s 3 risk drivers, CT, PCE, and TCE (see the authors’ Table 1, Table 2, Table 3, Table 4 and Table 5). PCE levels are four times higher than US EPA’s Maximum Allowed Contaminant Levels (MCLs) for drinking water [91].

*Third*, DTSC’s inductive experience suggests site-groundwater contamination. DTSC says “most sites” with chlorinated-VOC soils have groundwater “impacts” that require testing [62,92,93], partly because chlorinated-VOCs do not attenuate or become reduced, as petroleum-based VOCs do (according to the Johnson and Ettinger model). Thus, chlorinated VOCs can have far worse impacts on groundwater and indoor air than formerly thought—and than site documents claim [94] (p. 2ff.).

*Fourth*, the developer’s own site-geology data suggest groundwater contamination. Site soil is sandy, promoting downward migration of heavier-than-water solvents and DNAPLs [67] (p. 8).

*Fifth*, groundwater contamination is likely as the developer’s documents admit that higher- than-allowed levels of chlorinated solvents are “in all [site] areas and …depths” [86] (p. 26), from 0.15 to 46 m below ground surface, the deepest level tested [86] (p. 26); [71,93], at up to 744,000 times above allowed levels (see the authors’ Table 1, Table 2, Table 3, Table 4 and Table 5). Site-sampling thus confirms sandy-soil migration of VOCs. For all these reasons, this attempted justification, for the groundwater-data gap, fails.

#### 3.3.2. Justifying Groundwater Data Gap (8) by Promising Post-Construction-Groundwater Testing

State developers/regulators also attempt to justify the groundwater-data-testing gap by saying groundwater testing will be done post-construction, after onsite multi-story apartment buildings cover virtually the entire site. DTSC officials Ta and Hsieh say testing can be done later because “in-situ remedial technologies can be used to remediate subsurface soils and groundwater, even without physical access to the surface area over a release” [90] (p. iii).

However, such onsite, no-surface-access soil remediation would be difficult if not impossible because the site is highly and widely contaminated, and most soil sources of toxins have not been, and will not be, located/removed [67] (pp. 20, 28–29, 38–39); [70] (pp. 1, 8–10); [86] (pp. 31, 36, 44). Moreover, given the “widespread” extent/depth of the far-above-allowed levels of site contaminants [66,67] (see the authors’ Table 1, Table 2, Table 3, Table 4 and Table 5), source remediation likely would structurally compromise the newly constructed apartments, all built on contaminated soil [90] (p. 12).

In addition, the later-remediation argument does not justify pre-construction groundwater-data gaps because the site’s high levels of industrial solvents, with higher concentrations at deeper soil levels [67,71], strongly suggest DNAPLs, for which the site has never been tested. Experts agree that DNAPLs could not be located and removed post-construction, especially without full access to the area over the contaminants [95,96,97]. If not, pre-construction groundwater testing—to close data gap (8)—is needed to avoid precluding soil and groundwater cleanup, especially DNAPL cleanup.

#### 3.3.3. Gaps in Groundwater-Risk Data for CSM Area (4), Storm-Water Runoff

Because site assessors erroneously claim (see Section 3.3.1) there is no evidence of groundwater contamination, site documents have a data gap regarding (4) storm-water-runoff-contaminant risks to groundwater [86] (p. 7). This data gap seems serious and unjustified for at least four reasons.

*First*, site documents reveal above-allowed-level contaminants throughout the site, many within 6 inches of the surface [93]. Once the site’s asphalt cap is removed, these shallow-soil toxins could contaminate runoff. *Second*, some storm-water runoff migrates, recharging groundwater [98].

*Third*, site documents admit that “numerous” storm-drain samples exceed allowed contaminant levels [86] (p. 35), including for dioxins, polyaromatic hydrocarbons (PAHs), semi-volatile organic compounds (SVOCs), volatile organic compounds (VOCs), and metals (see the authors’ Table 1, Table 4 and Table 5). Although most storm drains have not been tested below 1.5 m [93,99], because site documents admit that storm-drain contaminants extend deeper than 9 m, contaminant-runoff has already migrated deep into soil, likely into groundwater [86] (p. 35).

*Fourth*, the storm-water-runoff, groundwater-data gap also seems serious and unjustified, as only 11 small, localized metals-hotspots will be removed before construction, leaving all other contaminants onsite [70] (p. 8); [86] (pp. 20, 29, 35), to contribute to groundwater risks from storm-water-runoff. As Section 3.3.1 showed, because surface contaminants already have migrated into storm-water drains and the deepest levels tested (45.7 m), storm-water could again contaminate site/groundwater. If so, storm-water runoff seems to be an unjustified data gap.

#### 3.3.4. Gaps in Groundwater-Risk Data for (5) Migration of VOCs

VOC migration to groundwater also is a key data gap for all the reasons given in Section 3.3.1, such as sandy soil, down-gradient well contamination, and contaminant-discovery at the deepest site-soil levels tested. Also, industrial solvents PCE, CT, and TCE are site “risk drivers” (see the authors’ Table 1, Table 4 and Table 5) [67] (p. 34); the greater their concentrations, the more likely their migration.

#### 3.3.5. Risk-Data Gaps for Ingestion, Inhalation, and Dermal Contact from Groundwater

Another groundwater-risk data gap concerns exposure routes of (13) ingestion, (14) inhalation, and (15) dermal contact. These data gaps are difficult to justify, as site groundwater could pose a health risk, given that wells very close to the toxic site are used for drinking water. In addition, groundwater/drinking water has been one of the most common exposure routes for PCE/TCE, as in Woburn, Massachusetts; Franklin, Indiana; Camp LeJeune, North Carolina; etc. (see Section 1).

As the US National Academy of Sciences warned, people are exposed to “VOCs in water by three major routes: inhalation, skin contact, and ingestion…showering, bathing, and drinking water” [100]. Because VOCs like TCE volatilize quickly in hot water, TCE exposure from a 10-min shower can cause risks as high as drinking TCE-laden water [101]. Such risks argue for closing the site data gaps for groundwater-risk exposure via ingestion, inhalation, and dermal contact.

#### 3.3.6. Data Gaps for Groundwater Ingestion/Inhalation/Dermal Contact Risk Harm to Children

The groundwater-ingestion/inhalation/dermal-contact data gap also is hard to defend, given no safe doses of PCE breakdown products like TCE and vinyl chloride; they cause immediate DNA damage at any dose. Their genotoxic carcinogenicity means that children—ages 2 and younger—face a 10-times-higher-cancer risk than adults, even from the tiniest dose [102,103]. For instance, the TCE drinking-water MCL is roughly 5 ppb [104], but US EPA warns that 0 is the only safe dose [5] (p. 7). The US Agency for Toxic Substances Control (ATSDR) warns that for “minimal risk” of permanent heart malformations, other birth defects, and immune-system harm, daily TCE-drinking-water intake should be 0.5 ppb TCE—10 times lower that what is allowed, and the daily inhaled/airborne dose should be no higher than 0.4 ppb—about 2 ug/m^3^ [5,102,105].

Yet there is no way to protect children from TCE, except through site preconstruction testing/remediation. Why not? As already shown, leaving a groundwater data gap precludes later soil cleanup, once buildings are onsite. Preceding sections also argue that the groundwater-data gap is serious, causing incomplete human-health risk assessment (see Section 3.4) [67] (the authors’ Table 3).

### 3.4. The Second, or Airborne Exposure-Route, Data Gap

Besides the groundwater-data gap, site documents reveal partial data gaps for CSM exposure route (12), inhalation, through (7) air, via (3) contaminant volatilization to soil vapor. This gap arises—as the health-risk calculation includes only indoor risks from VOC vapor intrusion—no other risks, as from ambient-air, soil-gas exposure. Yet carcinogenic-gas risks are extraordinarily high, up to nearly 750,000 times above allowed levels [67] (see the authors’ Table 1, Table 4 and Table 5).

#### 3.4.1. Airborne-Exposure Data Gaps for Outdoor/Ambient-Air VOCs

The assessors’ justification for ignoring soil-gas exposures—and leaving data gaps—is their claim that “VOCs in outdoor (ambient) air emanating from soil are not expected to pose a health risk” [67] (p. 31). Yet federal regulators clearly say (3) ambient soil gas presents health risks. Why?

*First*, the ambient-air-VOCs data gap in site assessments ignores the child-health alerts of the US ATSDR. ATSDR has repeatedly warned that ambient air is one of the two “most important routes of exposure” to TCE “for most members of the general population,” especially for “people living in the vicinity of a hazardous waste site” [5] (pp. 9, B-1). ATSDR likewise cautioned that in much of the US, TCE ambient-air levels can cause developmental harm, heart defects, central-nervous-system abnormalities, and other problems. It warned that because TCE can be absorbed from the atmosphere and concentrated in foods, “acceptable ambient air [TCE] levels may still result in food levels that exceed acceptable limits” [5] (pp. 9, B-1, 261, 334). Given such warnings, the ambient-air-VOCs data gap should be closed.

*Second*, the ambient-air-VOCs-data gap should be filled, given high VOC-soil-gas concentrations, up to nearly 750,000 times above allowed levels (see the authors’ Table 1). All other things being equal, the higher the site-soil-gas VOC concentrations, the higher the VOC-outgassing to ambient air [106]. Site source data also show that the outdoor/ambient-air, lifetime risk is as high as 1 cancer in every person exposed to only 2 of 11 VOC contaminants, PCE and CT. Once one considers the 9 additional VOC contaminants, whose concentrations exceed allowed levels, site cancer risks increase further—showing a data gap that should be filled [67] (see the authors’ Table 2).

*Third*, site-VOC levels present a health risk and data gap because, at other vapor-intrusion sites, outdoor-VOC concentrations are elevated above regulatory standards. Recall (from Section 3.3.6) that ATSDR says that to avoid permanent heart and other birth defects, airborne/inhaled TCE should be no higher than 0.4 ppb. [5]. Yet “mean TCE levels in ambient outdoor air across the US are generally between 0.01 and 0.3 ppb, although mean levels as high as 3.4 ppb have been reported” [5,107] (p. 9), and ambient-air VOC risks are often 65–700 percent higher than allowed levels [108,109,110]. Thus, US ambient-air TCE levels are extraordinarily close to what causes heart defects (0.4 ppb). This closeness argues for assessing onsite ambient-air TCE and other VOC risks, not ignoring them and leaving a data gap, as Pasadena-remediation documents have done.

*Fourth*, the outdoor/ambient-air, soil-gas data gap needs filling because most onsite VOC sources and DNAPLs have not been located and will not be removed [67] (pp. 20, 28–29, 38–39); [70] (pp. 1, 8–10); [86] (pp. 31, 36, 44). Yet just from the surface to 4.6 m, assessors have identified 17 different carbon-tetrachloride (CT) and PCE soil sources, scattered across the site [66,67] (see the authors’ Table 1, Table 4 and Table 5). Yet, only one of these carcinogen sources (in a metals-hot spot) will be removed. In addition, soil sources of CT and PCE that are deeper (than 4.6 m)—and sources for 9 other (non-PCE, non-CT) above-allowed-level VOCs, including TCE—have never been located, thus will not be removed [67] (pp. 20, 28–29, 38–39); [86] (pp. 31, 36, 44). Thus, they will outgas into ambient air for many years—just as non-removed sources and DNAPLs have done at other toxic sites [111,112,113,114].

If numbers of shallow-soil sources (up to 4.6 m below ground surface) of nine other VOCs are proportional to the 17 for shallow-soil CT and PCE (8.5 sources each, as preceding paragraphs show), then there should be 77 shallow-soil-VOC sources for nine other site VOCs for which no source-testing has been done. Likewise, if numbers of deeper-soil-VOC sources (up to 30.5 m) are proportional to those in shallow (to 4.6 m) soil (17 + 77 = 94), the Pasadena site should have up to 627 soil-VOC sources (100/15) (94) that will not be located or removed—a massive data gap, especially for child risks.

*Fifth*, the ambient-air-VOC-data gap also should be filled because elevated ambient-air-VOC concentrations influence indoor concentrations; contaminate much US-metropolitan-area ambient air; and likely cause continuing toxic-site-VOC outgassing. VOC releases are likely continuing because TCE has a half-life of 3–7 days in ambient air. Thus, TCE should not be present after about 50 days, unless there are ongoing releases. Yet because TCE continues to be present throughout the US, ongoing TCE releases must be occurring. These low releases are significant because many people are continuously exposed to them, leading to high population risks. Thus, apparently continuing releases of toxins like TCE (see the authors’ Table 5) should not left as a data gap [107] (pp. 1–2).

#### 3.4.2. Airborne-Exposure Data Gaps for Site Ambient VOCs: Special Risks to Children

Data gaps for ambient-air-VOC risks (see the authors’ Table 1, Table 2, Table 3, Table 4 and Table 5) are worrisome because, as noted earlier, VOCs are an important class of “air toxics” [16,17,18]—that are ubiquitous, particularly harmful to young children, and associated with many increased long-term health risks [11,108,115,116]. For instance, VOCs—especially site CT, dibromochloromethane, and TCE, three main site risk drivers [67] (p. 34), are strongly photochemically reactive. As ozone precursors, they react in sunlight with nitrogen oxides to form ozone or smog [117]. Yet ozone has no safe dose and is a leading cause of child asthma morbidity and mortality [118,119,120,121,122,123]. Three is the median age for asthma onset [119].

Compared to adults/older children, younger children take in more air, absorb more ozone, and can face permanent ozone-caused reductions in lung function. Emissions from VOC toxic sites, like this one, can even prevent their organs from developing properly [121,123]. VOCs also can travel for scores of kilometers, to cause deadly ozone. Because the Pasadena site’s air-toxics (such as CT, dibromochloromethane, and trichloromethane) contribute to already harmful Los Angeles County ozone/smog risks—assessors should close this ambient-air-VOC data gap [123] (pp. 4, 5, 11–13, 22).

#### 3.4.3. Airborne-Exposure Data Gaps, Especially for Particulate-Metals (PM) Risks to Children

The site-metals-dust data gap is likewise one that especially harms younger children. This is because, once the toxic-site asphalt cap is removed, windblown dust will transport toxic-site metals, carcinogens, and gases, putting children at risk. Four reasons argue for closing this site data gap.

*First*, the data gap for dust- or PM transport of heavy metals could be a significant because high levels of metals will remain onsite and can be blown for many kilometers, at least during the 3–4 years of site grading and construction, when site soils are exposed. Because preconstruction site remediation will remove only 11 small metals-hotspots [67] (pp. 20, 29); [70] (p. 8), sampling logs show that significant concentrations of above-allowed-levels of metals, like lead and mercury, will remain [71,93], such as at sampling locations USC-BC-126-01 and V2. USC-BC-126-01 has 15 times the allowed lead levels, 1170 mg/kg [71]. Because neither lead nor mercury has a safe dose, and both can cause permanent cognitive impairment in children [4], this data gap should be remedied.

*Second*, the site-metals-dust data gap should be closed because it could be even more serious than believed. Why? Most site-soil “sources” of metals likely have not been located, thus will not be removed, because site owners have not allowed sampling inside/below 68 percent of site buildings—roughly half of site-soil surface. These buildings could cover high soil-metals levels, because at least half of site buildings appear to have been used for metals testing/development/fabrication/cleaning—and to have had floor drains, into site soil [66] (pp. 24–32), [67].

Given limited building access, 10 of 11 named metals-hotspots are outdoors, where assessors always had site access. By mostly ignoring under-building, soil-metals testing, assessors have focused on easily assessed, easily removed, outdoor storm drains/pits [67]. If so, the site metals-hotspot designation may be a function—not of where the highest soil-metals concentrations are but, instead, a function of where testing was allowed (outdoors), and what is easy to detect and remove (storm drains/pits). If so, assessors should fill this metals data gap [66] (pp. 24–32), [67].

*Third*, the site-soil-metals-dust-transport data gap should be addressed because, despite the absence of a safe dose of many metals, many of them will not be removed, and their windblown dust will expose nearby neighborhoods. Yet any level of lead exposure to children during sensitive brain development, especially ages 2 and younger, risks lower IQ, neurological deficits, and psychological problems like aggressiveness and criminal behavior [124]. Something similar holds for mercury, another no-safe-dose site metal. Though some site-mercury concentrations—that exceed allowed levels—will not be removed (e.g., location V2), any mercury exposure risks IQ, developmental, cognitive, neurological, psychological, linguistic, immune, heart, motor and intelligence deficits in young children [125]. Both reasons suggest full metals testing is needed, to close this data gap [67].

*Fourth*, the soil-risk-data gap for dust-transport of heavy metals is significant and should be closed because the site’s extraordinarily high wind speeds could scatter windborne metals at least 5–31 km offsite. The average Pasadena-site wind speed is 21 km/h [126]—50 percent higher than what meteorologists classify as “high winds” (15 km/h and above) that can erode soil and blow dust high into the air [127]. In arid, drought-prone, southern California, “even moderate (wind) disturbance” causes such metals-dust transport [128]. Similar reasons explain why 22 tons of soil/year blow from the Sahara Desert into the Americas [127,129,130,131].

Moreover, the toxic-site’s high winds threaten its urban population on all sides. Pasadena has mountains to the North, desert to the East, and the Pacific to the West and South. Consequently, during every month of the year, 10–40 percent of total Pasadena wind comes from each of the four directions and buffets the city, including the 20,000 people—and 5000 children—who live within 2 km of the toxic site [132]. Once the site asphalt cap is removed, these daily high winds will widely transport metals-dust particles, a risk for which the data gap should be closed [133,134].

*Fifth*, the CSM data gap for metals-dust transport should be closed because site-screening levels ignore this risk. DTSC warns that its toxic-site screening levels exclude windblown- contaminant-dust risks [135] (p. 7), though they put young children most at risk. Thus, DTSC advises that “if [risk] pathways e.g., excessive dust generation,” occur at a toxic site, like Pasadena; if these standard screening levels are used; then the resulting “risk evaluation may significantly underestimate risk” [135]. Thus, site documents should remedy this windblown-dust data gap.

#### 3.4.4. Airborne-Exposure Data Gaps, Especially for VOC-Particulate-Matter (PM) Risks to Children

Another CSM-data gap concerns (2) transport of site VOCs that adhere to PM. Because the toxic site abuts the 10-lane, I-210 freeway, with its high PM levels; because high site winds will create significant PM/dust/gases, especially during years of earthmoving/construction; and because of site- outgassing VOCs (see the authors’ Table 1, Table 4 and Table 5), this PM will adsorb and absorb site VOCs [136,137]. Yet site documents calculate no PM risks. Four reasons suggest this gap should be closed.

*First*, site risk drivers, VOCs, will “significantly contribute” to “toxic health effects” of PM, especially for young children [138,139,140]. Because PM has no safe dose, and children’s lungs are still developing, even short-term exposure—hours or days—could cause permanent lung-development impairment [141,142,143,144]. This PM-VOC lung damage occurs because VOCs, adsorbed on particle surfaces, promote more inflammation than PM alone [137,145]. Yet VOCs, the most dangerous part of PM, can constitute about 60 percent of airborne-PM mass [146]. Because outgassing VOCs make no-safe-dose PM more harmful, especially for still-developing lungs, this data gap should be closed.

*Second*, the PM-VOC data gap also needs closing because VOCs are major precursors for urban fine PM formation, as already mentioned. They contribute to no-safe-dose PM and ozone/smog that especially harm children’s lung function [147] (p. 3833). As in Houston, Beijing, Mexico City, and Los Angeles County (where the site is located), VOCs (like CT, dibromochloromethane, and TCE) already are some of the worst primary pollutants [147]. Given the already-heavily-polluted location of the toxic site, there is special need to close this PM-VOC-data gap.

*Third*, the PM-VOC data gap, associated with site-outgassed VOCs, should be remedied because VOC-risk-data studies are feasible. For years, scientists have quantitatively assessed the increased human-health and smog risks associated with PM-and-adhering-VOCs [148,149,150,151,152,153,154].

*Fourth*, the PM-VOC data gap (see the authors’ Table 2 and Table 3), should be closed because site documents admit but fail to assess this problem, especially for vulnerable young children. Site documents admit that extremely toxic VOCs will “adhere to soil particles and become airborne contaminants,” a “health hazard,” whenever wind blows over uncapped or excavated site soil [86] (pp. 38, 67). Yet instead of calculating these PM-VOC risks, site documents say this risk “would be mitigated through air monitoring” and work-stoppage during high winds [86] (pp. 50, 63, 64). Yet because of the data gap, it is impossible to know whether planned mitigation is possible. After all, as Section 3.4.2. warned, work-stoppage during high winds does not stop the winds that blow exposed site contaminants. If not, this data should be closed, and its results used to re-assess site mitigation.

### 3.5. Site Data Gaps for Risks to Receptors, (16) Workers/Site Residents, and (17) Area Residents

A third data-gap area is risk to receptors/pollution victims. These include area/site residents, especially children—and site-adjacent school children and medical and emergency-care patients.

#### 3.5.1. Data Gaps for Schoolchildren and (17) Area-Resident Receptors

Because site documents calculate no risks to (17) area residents, especially children (see Section 3.5.4 below), they ignore their risks from breathing carcinogenic vapors and windblown site contaminants. Partly because Pasadena City College Extension abuts the north side of the toxic site (and admits dual-enrollment middle- and high-school students) [155], this data gap is significant. School buildings are less than 30.5 m from the toxic site. Yet DTSC says any buildings within 30.5 m of a subsurface toxic-gas plume—like the plumes under this entire site—should be evaluated for carcinogenic-vapor-intrusion [106]. Such subsurface carcinogenic gases can travel even farther than 30.5 m, even upgradient to the many homes near the site [156,157,158,159]. This is true because of the site’s sandy soils and high VOC concentrations—nearly a million times higher than allowed (see the authors’ Table 1, Table 4 and Table 5). Yet no offsite soil-gas testing has been done.

In addition, as already mentioned in Section 3.3.6, this child-risk-data gap should be closed because many site contaminants exceed allowed levels, are genotoxic carcinogens without a safe dose, and are 10 times more harmful to young children [102,105,160]. These genotoxins include TCE; benzo(a)pyrene, dibenz(a,h)anthracene, methylene chloride, benz(a)anthracene, benzo(b)- fluoranthene, chrysene, and indeno (1,2,3-cd) pyrene. Yet site documents have no mention of heightened, genotoxic risks to site-resident children and middle-, high-school, and college students at Pasadena City College. Instead site documents say only: “The 9.15 acre (toxic) site is bounded to the north by East Foothill Boulevard [86] (p. 1). Obviously, this data gap should be closed.

#### 3.5.2. Data Gaps for Risks to (17) Area Residents and Patients at the Adjacent Medical Facility

Site risk/remediation documents likewise have no risk data for (17) area residents—including children—and no data on site risks to medical patients, especially child, elderly, and urgent-care patients at the multi-story, hospital-size, Kaiser Permanente medical facility that abuts the east side of the toxic site. It covers an area roughly half the size (4.5 acres) of the toxic-waste site (9.15 acres). For at least two reasons, this risk-data gap for medical patients, especially children, should be closed.

*First*, the medical-facility buildings are less than 15.2 m from the toxic site—and only about 30.5 m from subsurface PCE, TCE and other “sources” [66]. As already mentioned, DTSC says anyone in buildings within 30.5 m of such subsurface, carcinogenic soil-gas plumes is in danger from vapor intrusion [63,106], and they should be tested. Yet, no offsite testing has been done.

*Second*, the site Health Evaluation assesses no carcinogenic-gas risks to adjacent medical patients/offsite receptors, despite DTSC’s warning of their high risks (see the authors’ Table 1, Table 2, Table 4 and Table 5) [63,106]. Site documents say only, “The 9.15 acre (toxic) site is bounded to the…East by a Kaiser Permanente medical facility” [86] (pp. 1, 2, 5), but this offsite-testing data gap should be closed.

#### 3.5.3. Data Gaps for Risks to Site (16) Construction Workers Who Are Receptors

Remediation documents likewise calculate no site risks to construction workers, despite their grading and bulldozing contaminants whose concentrations are nearly a million times higher than allowed (see the authors’ Table 1, Table 4 and Table 5). Instead, site documents say only: “potential risks include exposure of onsite workers to health and safety hazards typically encountered during construction activities and fugitive dust and VOC emissions during soil excavation activities” [86] (p. 55). This data gap should be closed, as already noted, as construction workers will be those most directly exposed.

The construction-worker data gap is especially obvious, as the Health Evaluation [67] (see the authors’ Table 2) assesses (i) no ambient-air risks for anyone (ii) no risks to vulnerable populations like construction workers, medical patients, children, and surrounding neighbors and (iii) no groundwater risks. To admit such serious risks, but neither calculate them nor inform victim populations is inconsistent, threatens public health, and precludes meeting safety standards.

#### 3.5.4. Data Gaps for Risks to Site-Resident Receptors Who Are (17) Children

However, the most serious data gaps concern child-resident risks. Although the main remediation document [67] calculates no child risks, its one-page CSM lists “child resident” as a toxic-site-risk receptor [89], yet excludes them from the Health Screening [67] (see the authors’ Table 3). The other site-remediation document [86] mentions only risks to children from onsite lead, but neither quantifies nor clarifies this risk. For instance, it never warns that lead has no safe dose, that any dose—especially to younger children—risks permanent brain damage, as Section 3.4.2 explained. Instead the document says only: “Lead is a COPC [contaminant of potential concern]. The main target for lead toxicity is the nervous system, both in adults and children” [86] (p. 28). At least six reasons argue for remedying this child-risk-data gap.

*First*, as noted in the introduction, most cancer victims, at most PCE/TCE toxic sites, are children. The younger the children, the greater their risks, as in Woburn, Massachusetts; Franklin, Indiana; San Francisco, California; and other PCE/TCE sites. Unless Pasadena assessors calculate site-resident-child risks, they may make the same mistakes that caused child cancers elsewhere

*Second*, the CSM resident-child-risk-data gap should be remedied because the main reason—that children are the first and disproportionate-in-number victims at hundreds of PCE and TCE sites—is that children are far more sensitive to genotoxic carcinogens than adults are. As Section 1 noted, because of this higher child sensitivity, US EPA directs risk assessors to use an ADAF [102,105,160]. However, because site assessors used no ADAF and calculated no risks to children, this is arguably a significant data gap; it violates US EPA’s ADAF directive.

*Third*, site-toxics risks to children should be calculated because young children have “only one chance” to develop a heart, brain, lungs, etc. When toxins harm them, they face permanent structural damage to bodily organs, not merely a curable medical ailment. Because treatment typically cannot correct their permanent developmental harm [102,105,160,161], this data gap should be closed.

*Fourth*, child-site-resident risks should be assessed because they are very high, and cleanup levels do not protect children from birth defects. As Section 3.3.6 noted, to avoid cardiac and other birth defects, US ATSDR says airborne/inhaled TCE doses must be no higher than 0.4 ppb/day—about 2 micrograms/cubic meter (ug/m^3^)/day [2,5,162,163]. Yet remediation documents allow beside-building, carcinogenic/mutagenic TCE soil-gas levels as high as 480 ug/m^3^—240 times higher than what causes permanent heart defects in children [67] (pp. 40–41), [162,163]. Given this apparent failure to protect site-resident children, their risks should be assessed, not left as a data gap.

*Fifth*, the developer’s preceding allowed TCE soil-gas levels—480 ug/m^3^ [67] (pp. 40–41)—are 1000 times less safe than mandated California and US-EPA cleanup levels of 0.480 ug/m^3^ TCE, given in site documents [71]. If the developer is not following state/federal cleanup standards, at least there should be no TCE child-risk data gap. People deserve to know what risks they may face.

*Sixth*, the developer’s allowed TCE soil-gas levels—within 5 feet of the surface, are 4250 ug/m^3^ [67] (pp. 40–41)—266 times less safe than the 2019 California-recommended soil-gas cleanup levels of 16 ug/m^3^ TCE [164]. Given the ADAF (Section 1), this 4250 ug/m^3^ TCE level will be 2,660 times less safe for children aged two and younger—than what Cal EPA and US EPA advise. Arguably site assessors should fill these gaps of risks to children. After all, children cannot protect themselves.

#### 3.5.5. Data Gaps for Risks to Adult Receptors Who Are (16) Site Residents

Site remediation and toxics-removal documents also have a data gap for risks to adult (16) onsite residents, another potential receptor/victim population. Instead of assessing all their site risks, from all contaminants through all pathways, remediation documents assess only VOC risks, only under four counterfactual conditions. These are (i) that one is exposed to VOCs only within 9 m of the surface [67] (p. 33), not within 30.5 m, as DTSC warns [63,106]; (ii) that one is exposed only to average (not the highest) VOC-soil-gas concentrations; (iii) that some technology can reduce this VOC-soil-gas risk by a *factor of 1000*; and (iv) that one is *indoors* and receives some assumed protective technology. There is no assessment of site-resident risks from non-VOC contaminants; from groundwater used for drinking water (Section 3.3); from outdoor carcinogenic gases—a million times higher than allowed (Section 3.4.1); and from windblown metals/toxins (Section 3.4.2–Section 3.4.3) [67], [70] (p. 8), [86] (p. 48), (see the authors’ Table 3). Two reasons argue for closing this data gap.

*First*, site documents admit that “COPCs [contaminants of potential concern] could remain in place at concentrations that may exceed some regulatory screening levels”; carcinogens will remain because the only required pre-construction removal is of 11 small metals “hotspots” [86] (p. 48). If contaminants remain, there should be no data gaps regarding their levels/risks; otherwise, cleanup might not meet regulatory standards, and site residents will lose their “right to know” these risks.

*Second*, remediation documents admit that site residents—especially young children—could face high cancer risks because no pre-construction carcinogen-remediation will be conducted, and any needed carcinogen-remediation must be done later, after thousands of residents are already living onsite. Site documents admit that “future residential and commercial users could be exposed to [carcinogenic] vapor intrusion until such time that soil vapor is removed which could be more than a year” [86] (p. 50), [67] (pp. 35–43). Because the developer has rejected preconstruction, site-carcinogen remediation, and because this risk is significant, this risk-data gap should be closed.

#### 3.5.6. Data Gaps for Risks to Receptors Who Are Area Residents, Especially Children

Site-remediation assessments/plans likewise have another major risk-calculation-data gap, for risks to (17) area residents. Four reasons argue for closing this data gap.

*First*, site-remediation data show that highly carcinogenic site soil—which will be graded and excavated in open air—has levels of industrial-solvents that are nearly a million times above allowed levels (see the authors’ Table 1, Table 4 and Table 5). Given such high levels of contamination, it begs the question to assume site-exposure risks from these carcinogens are low, then to allow cancer-risk-data gaps.

*Second*, as Section 3.4.1, Section 3.4.2 and Section 3.4.3 argued, site-remediation documents admit that because “inhalation of airborne [carcinogenic] dust particles” could occur [86] (p. 38), and because site toxins could “adhere to soil particles and become airborne contaminants” [86] (p. 67), state law requires site-monitoring for airborne-toxics risks, “to ensure that unsafe concentrations of dust are not migrating offsite” [86] (pp. 66–67). Yet if these risks require site-document warnings, they also require quantitative calculations of site risks to potential victims. Leaving this data gap both begs the question that windblown-toxic-dust-and-vapor risk is low and puts potential victims at risk.

*Third*, remediation documents admit that “inhalation of airborne dust and volatile chemicals in ambient air” poses risks, yet without evidence, claim that only site-construction workers face these non-quantified risks [67] (p. 31). Not quantifying this risk for all receptors again begs the question.

*Fourth*, the area-resident, dust-data gap should be closed because, during earth-moving and construction, site documents allow windblown contaminants to threaten 20,000 residents, including 5000 children who live within 2 km of the site. They allow above-allowed levels of windblown-contamination of offsite neighborhoods, at dust levels of 50 micrograms per cubic meter (µg/m^3^)” [86] (p. 68). Although the authors’ Table 6 shows such levels could jeopardize area residents’ health, the site Health Evaluation never included this, or any other, risks to children [67] (see the authors’ Table 3). Because site documents admit that higher-than-allowed-contaminant levels could harm people, they should quantify this risk, not beg the question.

### 3.6. Results of Assessing Possible Determinate Bias in the Data

To assess possible determinate bias or directionality in the preceding data gaps, the second DUE method followed three main steps: (1) identifying each CSM data gap; (2) using known site data to determine whether each gap is directional or not; and (3) assessing whether or not, overall, the gaps contribute to under/over/correctly estimating site risks. Subsequent paragraphs show that site-data gaps are unidirectional/determinate. They underestimate site risks, especially to children.

#### 3.6.1. Identified CSM Data Gaps

Results of the first step, (1) above, appear in preceding Section 3.3, Section 3.4 and Section 3.5. They revealed three potential data gaps—regarding *groundwater risks*, *airborne risks* from windblown PM and carcinogenic vapor, and *receptor or victim risks*, especially to children and area residents. The groundwater-data gaps exist because no site-groundwater-testing has been done. As shown earlier, the air-medium data gaps occurred mainly because there has been:

- no testing/assessment of outdoor/ambient-air contaminants;
- no human-health-screening assessment of most airborne site risks. (Site documents calculate only VOC risks, only from *indoor-air;* only by assuming a 1000-fold level of technological protection (not guaranteed for the site); and only after assuming *average*-VOC levels);
- no child risk assessment, despite the 10-fold-higher TCE cancer risks that young children face;
- no full soil testing (site-access problems caused half the site to be excluded from soil testing.);
- no calculation of windblown soil-metals risks, though many onsite-excess-metals areas (that exceed allowed levels) will not be removed; and
- no calculation of PM risks (from windblown PM gases and PM-adhering VOCs).

Similarly, Section 3.5 revealed that the site *victim/receptor data daps* occurred because there has been no calculation and assessment of risks to:

- sensitive groups on site-adjacent property, e.g., schoolchildren, medical/urgent-care patients;
- area residents, especially children, from illegal levels of windblown-site-soil metals/toxins/ carcinogens, though US ATSDR says wind is one of two main ways people are exposed to TCE;
- area and site-resident children, from developmental harm, birth defects, child cancer, and neurological harm, despite US EPA’s directing use of the ADAF to protect children.

In short, site-remediation documents reveal data gaps regarding groundwater-media, air-media, and pollution-receptor risks. Are these data gaps merely problems of quantitative imprecision, or do they reveal directionality, a systematic bias in data collection?

#### 3.6.2. Determining the Overall Directionality, If Any, of Data Gaps

Given the preceding data gaps, do they show any directional bias, in terms of supporting/not supporting the null hypothesis, that is, no effects from site data gaps? Consider the directionality of these three risk-data gaps, regarding groundwater, airborne contaminants, and potential victims/receptors.

Section 3.3 showed that site-remediation documents contain no quantitative-risk information about contaminated-storm-water runoff and soil-contaminant migration (especially of carcinogenic-industrial-solvent VOCs) into groundwater. As a result, there is no quantitative risk assessment of *groundwater threats* to potential victims/receptors, through ingestion, inhalation, or dermal contact.

Section 3.4 likewise revealed that site documents contain no quantitative-risk information about *threats from inhalation of airborne-contaminant* PM and carcinogenic gases, by residential and area receptors/potential victims.

Section 3.5 revealed that site-remediation documents contain no quantitative-risk information regarding *threats to key potential victims/receptors*. These include people who are outdoors, within several kilometers of the site, especially children; site construction workers; and adjacent schoolchildren and medical patients. Other victims include those who are indoors, in nearby homes, the adjacent school, or the large medical facility—who could be victims of offsite carcinogenic vapor intrusion. DTSC requires such offsite-vapor testing [106], but this has never been done.

Each of these (Section 3.3, Section 3.4 and Section 3.5) data gaps is biased toward the null or no-effect hypothesis, because each gap represents dangerous and ignored effects—as having a risk value of zero. This is because none of these omitted risks is included in the human-health screening evaluation, which is supposed to sum all types of risks, via all exposure pathways. By omitting these risks, the resulting, supposedly aggregate, risk is incomplete, an underestimate. For young children, for reasons already given, these risk-underestimates are likely as much as 10 times greater than adult risks.

#### 3.6.3. Assessing Whether These Data Gaps, Overall, Contribute to Over/Underestimates of Site Risks

The previous section showed that each CSM data gap-outlined in Section 3.3, Section 3.4 and Section 3.5—likely imposes risks on potential victims. Yet site documents ignored these risks, thus counted their harm as 0 because the risks are ignored, unquantified, and unanalyzed.

Yet, each omitted risk is likely greater than 0, partly because each is part of the CSM risk factors. Each omitted risk also is likely greater than 0 because regulators such as ATSDR [5,6]; DTSC [92,106,135]; and US EPA [63] have repeatedly and forcefully warned against ignoring these important pathways of harm at toxic sites. For instance, they have warned against data gaps regarding special toxic-site threats to children, e.g., [5,63]; airborne carcinogenic-soil-vapor risks to area residents [5]; carcinogenic-soil risks to groundwater [63,106]; and so on. That is, the data gaps show that site assessors have not followed the mandated government rules for toxic-site assessment and cleanup. Instead they have taken shortcuts that ignore many risks, thus leave data gaps.

Moreover, there is no foreseeable way that these risks—all counted as 0 and thus all supporting the null or no-effect hypothesis—could be health-positive. Why not? All the ignored risks or data gaps involve effects of exposure to some of the most toxic and carcinogenic chemicals known, like TCE. Because none of these ignored health risks/effects could be health-positive, because government regulators specifically warned against ignoring these risks, yet because site assessors counted all these toxic risks as zero, overall, these data gaps are unidirectional. They underestimate site risk. Therefore, they provide evidence of determinate bias.

## 4. Discussion

Given the preceding evidence for determinate scientific bias in the Pasadena-toxic-site-remediation documents, at least six questions come to mind:

1. Why did site assessors not calculate groundwater risks, when they have been a main exposure route for TCE and PCE at other sites where they have caused widespread cancer?
2. Why did site assessors not calculate airborne/soil-borne site risks—the main site risk drivers?
3. Why did city officials and state regulators not find and correct these data-gap problems?
4. Why did Pasadena officials not do a better job of protecting city residents, especially children?
5. What future research might promote further analysis of DUEs to deter flawed toxics cleanup?
6. What general principles does this case study suggest for improving toxic-site remediation?

### 4.1. First Question: Why Was There No Calculation of Groundwater Risks, a Key TCE/PCE Exposure Route?

Site assessors attempted to answer this question in site documents. Consider their six responses.

#### 4.1.1. Is “No Information” Known about Site Groundwater, as Assessors Claim?

*First*, site assessors said that “No information is currently known about groundwater at the site, therefore the HHSE [Human Health Screening Evaluation of site risks] cannot take groundwater into account at this time” [67] (p. 35). However, these data are unknown because the developer chose to do no preconstruction groundwater sampling. Moreover, the assessors’ own documents admit that site contaminants have been found in site-adjacent drinking-water wells, beginning before 1994 [67] (p. 6). Hence it does not appear true that “no information” is known about site groundwater.

#### 4.1.2. Are Nearby Well Data Unavailable, as Assessors Claim?

*Second*, site assessors attempted to explain their not performing groundwater-risk assessment by saying “We have not been able to obtain recent analytical data from the three wells” near the site [70] (p. 11). However, as earlier sections of this analysis noted, the closest state-groundwater- monitoring well (well 1), 2 km down-gradient from the site, is contaminated with the three main site risk drivers, CT, PCE, and TCE. Its PCE levels are 4 times higher than the US EPA MCL [91]. The senior author obtained these well data by logging into the state groundwater-information system, setting up a password, and downloading the data. Thus, it is false that the data are not available

#### 4.1.3. Is Preconstruction Site-Groundwater Testing Unnecessary, as Assessors Claim?

*Third*, assessors attempted to explain their failure (to perform groundwater testing) by claiming it was unnecessary, given that depth to groundwater is greater than 91.4 m, and groundwater is not “a planned source of drinking water” [67] (p. 31). However, DTSC clearly said, in site documents, (1) that there are two active drinking water wells within half a kilometer of the site [70] (p. 7). and (2) that: “The water under the site is designated for beneficial use…The property owner does not own the water rights under this site, which is located in an adjudicated basin. Considering all the facts above, groundwater must be included” in site risk assessments [70] (pp. 8, 10). Moreover, the assessors’ own data show that soil is sandy and that depth to groundwater is 58.2 m in some places, not greater than 91.4 m [67] (pp. 5, 6), as the assessors claim.

In addition, US EPA and DTSC documents show that vapors and drinking water from contaminated groundwater—hundreds of meters below residences—could still cause cancer in those residing nearby at the surface; this is especially because of (1) soil contamination, nearly a million times higher than allowed (see the authors’ Table 1, Table 4 and Table 5), and (2) the sandy, rocky, site soils through which carcinogenic gases can move quickly [63,70] (p. 10). That is why, in site documents, DTSC scientists told the Pasadena developer/assessors to provide “evidence that the deep (carcinogenic) soil-vapor concentrations proposed to be left in place will not be a future threat to groundwater (and to) develop site-specific soil-vapor remediation goals protective of ground water” [70] (pp. 10, 20). However, assessors have not done what DTSC scientists asked; most site soil has not been tested, and assessors will remediate only the top 9 m of soil at the site, not everything above groundwater [67] (pp. 41–42). Thus, contrary to the assessors’ third argument, groundwater testing appears necessary.

#### 4.1.4. Would Post-Construction Testing Protect Groundwater and Receptors, as Assessors Claim?

*Fourth*, site assessors attempted to justify their failure to do preconstruction-groundwater-risk assessment by committing to do a full year of post-construction-groundwater testing [67] (p. 5), [70] (p. 12). However, later testing will not work because preconstruction tests are needed, in part, to reveal what site-soil toxins/sources are contaminating groundwater, so that these soil-contaminant sources can be removed, before site development [63]. This removal is necessary, as DTSC says, to ensure that “soils are not a continuing source of groundwater contamination” [70] (p. 9).

In addition, contaminants cannot be removed, post-construction, as the assessors claim, because even limited site testing and isoconcentration maps (for only 2 of the scores of contaminants) cover at least one-fourth of the site [66,67], and their removal would undermine the developer’s multistory apartment buildings [72]. US Superfund regulations (required for this site), including US EPA Guidance on assessing soil-vapor sites [63], like this one, mandate preconstruction groundwater testing/cleanup, as does the required National Oil and Hazardous Substances Pollution Contingency Plan [165,166]. Thus, the assessors’ plan for later groundwater testing will not work.

#### 4.1.5. Is Post-Construction Site-Groundwater Testing Essential to Development, as Assessors Claim?

*Fifth*, assessors tried to explain not performing preconstruction, groundwater testing/assessment by claiming that because it would interfere with site construction/earthmoving, it must be done later [67] (p. 32). However, this response begs the question. Predevelopment groundwater testing will not interfere with earthmoving. This response suggests the developer gives groundwater testing (and public health/safety) second priority, after site-construction, rather than giving health/safety first priority, and site-construction second priority. Should public health/safety not come first?

The developer/assessors admitted their questionable priorities in site documents. They said later groundwater-testing wells must be placed, not in areas where scientifically rigorous sampling requires placement, but where they “will not impact planned site development” [70] (p. 7). They likewise said preconstruction removal of soil-based groundwater contaminants should not be done because it “is not a timely means of remediating the site once site development is approved to proceed” and would interfere with the developer’s planned rapid site construction [70] (p. 10). However, this reason fails, as it provides no *scientific* justification for failure to assess groundwater.

#### 4.1.6. Is Post-Construction, Site-Groundwater Testing Legally Defensible, as Assessors Claim?

*Sixth*, the site developer tried to explain not performing preconstruction-site-groundwater testing by claiming that the Prospective Purchase Agreement (PPA), that Trammell Crow obtained from California DTSC [167], did not require preconstruction groundwater testing, as it is “outside the responsibilities and obligations” of Trammell Crow [70] (p. 10). PPAs are state contracts that give toxic-site remediators-developers liability protection against future harm from site toxins, provided they are “willing to cleanup contaminated sites” [168]. This Trammell Crow response is correct, insofar as the PPA itself does not require preconstruction groundwater testing.

However, the PPA does not change the fact that state and federal cleanup rules require groundwater testing/assessment (see preceding paragraphs). Moreover, the PPA itself requires that “intended uses of the site (and all activities anticipated to be undertaken in connection therewith) will not pose health risks to persons,” and that the developer will “reduce the human health risk to acceptable levels.” Yet if previous arguments are correct, closing the groundwater-testing gap is necessary to meet both PPA requirements [167] (pp. 2, 5). In addition, the PPA says the state “may specify such additions, modifications, and revisions” to planned remediation, “as deemed necessary to protect human health and the safety of the environment” [167] (p. 8). Hence, to provide a scientific justification for not conducting preconstruction groundwater testing, Trammell Crow must show this failure or data gap nevertheless protects “human health and the safety of the environment.”

Moreover, if the DUE is correct, there is strong evidence that the PPA must be revised. One reason is that, for the last decade, a multi-committee group of state legislators has tried and failed to reform DTSC. In 2019, this DTSC-oversight, super-committee concluded that California DTSC.

- continues to have “an inadequate and unresponsive regulatory program,” especially problems with “transparency, accountability, and …. cleanup,” despite legislative “statutory changes… to help DTSC better achieve its mandates.” This has caused “decreased… public trust in DTSC”;
- “is not properly enforcing state and federal law and is allowing … numerous violations of state law and regulations… [and use of] outdated technologies, practices, and safeguards… [that] are potentially releasing hazardous wastes into the environment” [169] (pp. 7, 4–6).

Given these California-Legislature conclusions, the Pasadena developer may have induced DTSC (see the next section) to ignore state/federal toxic-cleanup standards. However, as Section 4.1 helps argue, this does not make either site data gaps or cleanup violations scientifically defensible.

### 4.2. Second Question: Why Did Assessors Ignore Site Risk Drivers, Not Test Most Air- and Soil-Borne Risks?

At least two possible explanations come to mind for the second, or airborne-risk, data gap. One reason may be that because no one did a DUE for site-remediation documents, the airborne/soil-borne data gaps were not obvious. The documents did claim that sampling data collected in 2006 were “usable for their intended purposes” because they met data-quality criteria such as adequate sample preservation and detection limits [67] (Attachment C, p. 3). However, no assessors ever did a DUE to assess the CSM completeness of the entire site-data set, as dictated by US EPA and some state guidelines [74] (pp. 25–42), [85] Likewise, no one has done a DQA for site data collected since 2006.

Why did no site assessors follow the US EPA guidelines for data-usability? One reason may be that DTSC has never mandated DUE evaluation, as other states (like New Jersey) do [85]. DTSC also has not developed its own version of the 1992 US EPA guidelines for data-usability evaluation [74], as states like New Jersey have done. Thus, in part, DTSC omissions may help explain the absence of a DUE for site-remediation documents, and thus the failure to ensure that documents had no data gaps.

Perhaps another reason Pasadena-remediation assessments have air/soil-borne data gaps is that such gaps are common in toxic-site cleanups, especially wherever DUEs are not required. For instance, Trammell Crow is the largest US commercial real-estate developer, with assets of more than $65 billion [69]. Its “Brownfields Acquisition and Development” division, to which it refers by the acronym BAD, is dedicated to cheaply purchasing and redeveloping toxic-waste sites [170,171]. For its BAD properties, Trammell Crow promises the public and city officials full cleanup, a safe site [172] (p. 2), [173]. However, after purchasing these properties, Trammell Crow often appears to allow the same questionable data gaps, especially regarding groundwater, deeper soil, and carcinogenic soil gas. Hence Trammell Crow’s Pasadena-site data gaps may reflect its wider corporate practices [84].

Another possible explanation for Trammell Crow’s data gaps is financial conflicts of interest. Since at least the 1980s social-science research has clearly established “the funding effect,” that research conclusions are likely to serve the interests of those who fund the research, an effect that is statistically correlated with research outcomes. Thus, if private companies fund research on safety of their chemicals, drugs, or site remediation, the research is likely to show safety. If nonprofit, university, or government agencies fund the same research, it is much less likely to show safety. This “funding effect” has been overwhelmingly established in environmental-health, tobacco, climate-change, chemical-toxicity studies, and so on; this effect also could be occurring in Pasadena toxics-remediation assessments by those whose profits are at stake [16,174,175].

### 4.3. Third Question: Why Did City or State Regulators Not Stop Site-Remediation Data Gaps?

Pasadena city officials were the first to approve site-remediation documents, but at least three factors could have encouraged their approving almost any housing development—even on an inadequately assessed toxic site. These three factors are California’s housing shortage [176], Pasadena’s $2-billion budget deficit and, thus, Pasadena’s needed tax-base expansion [177] (p. 11).

Another factor in the approval of scientifically deficient toxic-site-remediation assessments is that the Pasadena mayor (and developer) Terence Tornek and many city-council members accept campaign donations from developers—whose local projects they vote to approve/disapprove [178]. However, other cities, like Los Angeles, prohibit such donations [179]. Any of the preceding factors could have led city officials to blindly trust Trammell Crow’s safety promises [172] (p. 2), [173]. In addition, as Section 4.1.6 shows, because the state says California DTSC has not properly enforced toxics-cleanup for at least 10 years, the Pasadena site-data gaps may be part of a larger problem [84].

### 4.4. Fourth Question: Why Did Assessors/Officials Not Give Residents, Especially Children, More Protection?

Preceding sections revealed at least 7 reasons that children (ages 2 and younger) are most at risk from data gaps in Pasadena toxic-site-assessment/remediation documents. These younger children:

- Face up to *10-times-higher cancer risks* (than adults will) from the toxic site, yet assessors performed *no child risk assessment* to quantify/assesses any of these higher risks (Section 3.5.5).
- Face 10-times-higher cancer risks than adults from *carcinogenic-vapor intrusion*, especially from TCE, a genotoxic carcinogen having no safe dose (Section 3.3.6 and Section 3.5.1).
- Face *windblown*, *ambient-air TCE* dust/gas whose levels are 1040 times more harmful than the best California standards dictate (Section 3.5.6).
- Face *permanent heart defects* because TCE-soil-cleanup levels of 480 ug/m^3^ are 240 times less safe than the 2 ug/m^3^ TCE—that causes such birth defects in children (Section 3.5.4).
- Face *IQ losses* from windblown, no-safe-dose lead that will not be cleaned up (Section 3.4.3).
- Face *asthma and lung-function losses* from windblown carcinogens and PM (Section 3.4.4).
- Face *asthma and lung-function losses* from site VOCs that cause no-safe-dose ozone (Section 3.4.2).

#### 4.4.1. Assessors May Not Realize How Sociocultural Factors Put Children at Higher Toxics Risk

Given these heightened toxic-site threats to children, assessors may have failed to protect children because they tend to be natural scientists/engineers, thus unaware of child-health threats. Public officials also often trust engineering experts, not realizing they have little pediatric expertise.

In particular, assessors/officials may not know that specific social problems put children at greater risk from toxic sites. These already-noted problems include the facts that disproportionate numbers of vulnerable poor people, minorities, and children live near toxic-waste sites [2,180,181]; 25 percent of all US children—mostly poor and minority children—live within 2 km of toxic-waste sites [2,76,77,78,79,180,181]; most toxic-waste sites are in dense, urban areas [180]; and people living near toxic-waste sites have (statistically significant) higher rates of health harm [3].

For instance, children conceived by mothers living within 3 km of an unremediated toxic-waste site face statistically-significant more harm, compared to children—born to the same mothers—but not conceived when their mothers were living within 3 km. Compared to their siblings, near-toxic- site children are 10 percent more likely to be diagnosed with a cognitive disability; 7 percent more likely to repeat a grade; more likely to have 6 percent (of the standard deviation) lower standardized test scores in grammar school; and 7 percent more likely to be suspended from school [3,4,182].

#### 4.4.2. Assessors and Regulators May Not Realize That Children Are Not Merely “Little Adults”

Because few people are likely aware of the preceding statistics, they may think children are merely “little adults,” with the same biological vulnerabilities as adults. Yet as Section 1 outlined, children often are more biologically vulnerable to pollutants than adults, receive higher doses, and have a longer lifetime during which resulting harm can be expressed [180,183]. For all the preceding reasons, children are part of the roughly 25 percent of the population that is especially sensitive, including pregnant women and the elderly [184].

Yet in spite of children’s medical vulnerabilities, many regulations do not adequately protect children from toxic chemicals [185], ionizing radiation [186], or other contaminants. Instead, regulations often protect “reference man” [186], the white adult male, the most common medical-research and clinical-trials participant. (Experiments on children are not allowed, a fact that encourages less protective regulations for children.) As a result, people may not realize that children have “only one chance” to develop a brain, heart, or lungs; if toxins jeopardize this “one chance,” the resulting harm is often permanent [161]. Because children have “only one chance” [161] to develop, and often are 10 times more vulnerable to toxins, the US National Academy of Sciences/US Institute of Medicine, recommends “separate risk assessments for children and adults” in high-risk situations like toxic-site assessment/remediation [187]. This recommendation is consistent with US EPA’s requiring use of ADAFs (see Section 1) to assess risks to children [5,188]

### 4.5. Fifth Question: What Future Research Might Investigate DUEs as Ways to Deter Faulty Cleanups?

This article performed a focused or limited-scope DUE for the Pasadena-toxic-site-remediation documents. One useful area of future DUE research would be to perform a focused DUE for Pasadena remediation documents that addresses other site contaminants besides chlorinated solvents such as PCE and TCE. For at least three reasons, it would be important to perform a DUE that addresses soil levels of site polyaromatic hydrocarbons (PAHs), such as benz(a)anthracene, benzo(a)pyrene, benzo(b)fluoranthene, and dibenz(a,h)anthracene. One reason is that some of these PAHs far exceed allowable levels. A second reason is that some of them are genotoxic carcinogens, thus especially dangerous for children, including benz(a)anthracene, benzo(a)pyrene, benzo(b)fluoranthene chrysene, dibenz(a,h)anthracene, and indeno(1,2,3-cd)pyrene. A third reason to do future DUEs to address Pasadena-site PAHs is that many of them are semi-volatile organic compounds (SVOC) that are implicated in vapor intrusion, just as PCE and TCE are. Still another area of useful research would be to conduct a full-blown DUE for the Pasadena site, to determine whether other aspects of site-remediation documents exhibit similar problems with data usability.

An additional area of important research would be to determine whether remediation documents for other toxic sites also have CSM data gaps that result in site-risk underestimation/determinate bias and therefore are likely to cause less protective cleanups. If other sites exhibit these data gaps and determinate bias, as the authors suspect, evidence from these other sites will help strengthen the preliminary conclusion of this article, namely, that routine, independent DUEs may be needed to help deter flawed PCE/TCE cleanups.

### 4.6. Sixth Question: What General Principles Does This DUE Suggest for Improving Toxic-Site Remediation?

The results of this Pasadena DUE case study suggest that without routine, independent DUEs, site-remediation documents may fail to have complete, usable data needed for reliable cleanup. Thus, if this article’s results are generalizable, they suggest several important principles:

1. Safe toxics cleanup may require *implementing* the highest data-quality/usability standards.
2. Safe toxics cleanup may require *enforcing* the highest data-quality/usability standards.
3. Safe toxics cleanup may require *mandating* routine, independent, data-quality/usability controls.
4. Safe toxics oversight may require *prohibiting* officials’ discretionary acceptance of flawed data.

Principle 1 is important because, as Section 4.2 notes, most states—except for those like New Jersey—have not implemented the 1992 US EPA DUE guidance. California, for instance, has no document that implements US EPA DUE guidance. Yet this article shows how important DUEs can be, as one way to help ensure adequate testing and assessment data to support reliable site remediation. Yet guidance that is *not implemented* by states cannot guide state cleanups. Because both Pasadena city officials and the state DTSC approved the Pasadena toxic-site-remediation documents, despite their having numerous data gaps that jeopardize site safety, Principle 1 is important.

Principle 2 is important because although federal and some state authorities have excellent guidelines for toxic-site scientific-data audits, DQAs, DUEs, e.g., [74,85,87], these guidelines often are *not enforced* by state or federal authorities (see Section 4.1.6). Section 2 and Section 4.2 show that DTSC did not enforce federal and state DQA guidelines. As a result, both the city and the state approved the Pasadena site-remediation documents although the Pasadena DQA covers only some 2006 data, no data for the last 13 years, and no data generated by the current site assessors/developers. Yet valid DQA is a necessary condition for usable data and for doing a complete DUE [74,85,87].

Principle 3 is important because it recognizes that when routine, independent (third-party), data-quality/usability evaluation is *not required*, as at the Pasadena site, then public health and safe cleanup can be jeopardized. Given repeated failure to implement and enforce data-quality standards, routine, independent-party, data quality/usability assessment must be *required* for all toxic sites, particularly those in which polluters/developers do the remediation, as they have obvious financial conflicts of interest. After all, if site assessors/remediators/developers knew that their analyses would undergo routine data-quality evaluation by an independent third party, this knowledge would likely deter the sort of data gaps and risk underestimation that this article has revealed.

Principle 4 is important, as many toxics’ regulators, like the California DTSC, have apparent authority to use “discretion” to ignore state/federal recommendations for data quality/usability. [169]. If this exercise of discretion by regulatory agencies is a problem in other states, then protecting the public, especially children, from toxic wastes may require both routine, independent DUEs/DQAs/etc. and also prohibiting discretionary actions that allow violations of data-quality standards. Why? Data quality/usability standards—that are merely discretionary—are not really standards.

## 5. Conclusions

The preceding data-usability evaluation (DUE) shows three major conceptual-site-model, scientific-data gaps in the Pasadena toxic-site-remediation documents, namely, data gaps regarding:

- all risks from groundwater;
- airborne risks from windblown PM, carcinogenic vapors, and vapor intrusion;
- risks to many area receptors/victims, especially to onsite-resident and area children.

Because these gaps appear to represent determinate biases that cause underreporting of site risk and needed remediation, Pasadena-remediation assessments fail this focused DUE and are likely to lead to flawed toxic-site cleanup. While many Pasadena residents could be hurt by deficient toxic-site remediation, the authors showed seven distinct reasons that children will be hurt most (Section 4.4).

The preceding results are important because they suggest one reason for flawed PCE and TCE remediation, namely, failure to perform routine, independent DUEs. If the results of this article prove to be generalizable, they suggest that routine, independent DUEs might help to (1) prevent PCE/TCE health harm; (2) provide an economical early-warning system for potentially flawed toxic-site cleanups; (3) deter scientific fraud in toxics remediation; (4) ensure more timely waste remediation; and (5) reduce legal and financial liability for toxic-waste sites.

## Figures and Tables

**Table 1:** Onsite Perchloroethylene (PCE): Up to 5 Orders of Magnitude above Allowed Levels [67,71].

Sample Location Identifier	PCE (ug/L) Concentration	/0.00046 ug/L (Screening Level) = Times above Allowable Limit	To Be Removed?
NMSV10-5	342	743,480	yes
V9-15	137	298,000	no
VD2-30	122	265,217	no
V-5-15	79	172,000	no
V9-10	39.1	85,000	no
V10-5	36.3	79,000	no
NMSD3-60	22.3	48,480	no
V6-15	20.5	45,000	no
VD1-20	20.4	44,347	no
NASD3-113	17.9	38,913	no
V2-15	16.7	36,304	no
NMSV12-15	14.5	31,522	no
NMSV15-15	14.2	30,870	no
NMSV11-15	13.5	29.348	no
V18-15	13.5	29,348	yes
NMSV14-15	11.6	25,217	no
VD1-30	10.8	23,500	no
V8-15	10.5	23,000	no
NMSV2-15	10.2	22,174	no
V2-5	9.47	21,090	no
V18-5	8.32	18,090	no
NMSV13-5	5.51	11,978	no
NMSV4-15	1.29	2804	no

All Ninyo and Moore’s perchloroethylene (PCE) samples violate allowed levels, including the 25% of samples listed above [71], yet only two of these PCE locations will be removed, as they are in metals hotspots, the only areas to be removed.

**Table 2:** Cancer Risk from Site Industrial Solvents, Outgassing into Ambient Air [67,71].

VOC Carcinogen ^1^	Maximum in Soil Gas, ^1^ µg/L	Screening Level ^1^ (SL), µg/L (10^−6^ Risk)	(Maximum in Soil Gas ÷ SL) ^2^ = How Many Times above Government Limits?	Excess Cancer Risk ^2^
1,1-Dichloroethane ^3^	1.56	0.0018	867	8.67 × 10^−4^
1,1-Dichloroethylene	1.95	0.073	27	2.67 × 10^−5^
1,1,1-Trichloroethane	0.063	1	0	6.30 × 10^−8^
cis-1,2-Dichloroethylene	2.53	0.0083	305	3.05 × 10^−4^
Carbon Tetrachloride	28.4	0.000067	438,801	4.39 × 10^−1^
Chloroform	1.12	0.00012	333	9.33 × 10^−3^
Dibromochloromethane	0.998	0.00013	7677	7.68 × 10^−3^
Dichlorodifluoromethane	9.32	0.1	93	9.32 × 10^−5^
Trichlorotrifluoromethane	17.6	31	0	5.68 × 10^−7^
Tetra/Perchloroethylene (PCE)	342	0.00046	743,478	7.43 × 10^−1^
Toluene	0.244	0.31	0	7.87 × 10^−7^
Trichloroethylene	8.59	0.00048	17,896	1.79 × 10^−2^
Trichlorofluoromethane	14.7	1.3	11	1.13 × 10^−5^
Xylenes, total	0.718	0.1	7	7.18 × 10^−6^
Given lifetime exposure, TOTAL = certainty of cancer = 1				

^1^ Contaminant list, maxima in soil gas, and screening levels are from [67]. ^2^ To determine excess cancer risk, Department of Toxic Substances Control (DTSC) mandates dividing the maximum contaminant level by the screening level (1.0 × 10^−6^, a one-in-a-million cancer risk)—and then multiplying this ratio by 1.0 × 10^−6^ [67]. ^3^ For the majority of site industrial solvents (VOCs), that were tested using adequate detection limits, *all samples* (those not shaded above) violate allowed levels of the contaminant.

**Table 3:** Human-Health-Risk Calculations in Remediation Documents [67,71].

	Cancer from Indoor-Solvent-Gases	Cancer from Ambient-Air Solvent-Gases	All Threats, Ambient Air PM	All Threats, Drinking Water, Groundwater	Birth Defects, Developmental Disabilities
Adult site resident	Cancer risk = 1 in 2900 ^1^	No risk calculation, but risk = 1, certainty ^2^	No risk calculation	No risk calculation	NA
Adult site worker	No risk calculation	No risk calculation ^3^	No risk calculation	No risk calculation	NA
Adult area resident	No risk calculation	No risk calculation	No risk calculation	No risk calculation	NA
Child site resident	No risk calculation ^4^	No risk calculation ^4^	No risk calculation	No risk calculation	No risk calculation
Child area resident	No risk calculation ^4^	No risk calculation ^4^	No risk calculation	No risk calculation	No risk calculation

^1^ Ninyo and Moore [67] calculation for adults. ^2^ The authors’ calculation, in preceding Table 2, is for adults. ^3^ This risk is high for site construction workers, given no pre-construction carcinogenic-solvent removal. ^4^ This risk could be 10 times higher for children ages 2 and younger, than adults, given the age-dependent adjustment factor (ADAF) [5]—which must be applied to all genotoxic carcinogens such as trichloroethylene, benzo(a)pyrene, dibenz(a,h)anthracene, methylene chloride, benz(a)anthracene, benzo(b)fluoranthene, chrysene, indeno(1,2,3-cd)pyrene.

**Table 4:** Carbon Tetrachloride (CT): Up to 5 Orders of Magnitude above Allowed Levels [67,71].

Sample-Location Identifiers	CT (ug/L) Concentration	/0.000067 ug/L (Screening Level = Times above Allowable Limit	To Be Removed?
NMSD3-113	28.4	424,000	no
NMSD3-84	24.3	363,000	no
NMSD3-150	20.6	307,463	no
NMSD3-150	18.5	276,119	no
NMSD2-150	13.2	197,015	no
NMSD2-130	12.9	193,000	no
NMSD2-150	9.83	146,700	no
NMSD3-60	8.39	125,224	no
NMSO1-85	7.53	112,388	no
NMSD1-99	5.95	90,806	no
NMSD2-63	2.67	40,000	no
VD1-30	2.27	34,000	no
NMSD2-130	2.27	33,881	no
NMSV7-5	1.82	27,164	no
VD3-20	1.45	21,642	no
VD3-30	1.42	21,200	no
V2-5	1.39	21,000	no
NMSV6-5	1.38	20,600	no
V8-15	1.36	20,300	yes
VO12-15	1.19	18,000	no

60% of the 101 Ninyo and Moore [94] carbon tetrachloride (CT) samples violate US Environmental Protection Agency (EPA) allowed CT levels, and 40% of Ninyo and Moore [94] CT samples used tests that were 3000 times too lenient to detect effects higher than allowed levels. The values above represent 30% of the CT samples known (through properly sensitive tests) to violate US EPA CT allowed levels. Most illegal levels of CT will not be removed because they are not in one of the 11 small, localized metals-hotspots [67,70], the only spots onsite to be removed.

**Table 5:** Onsite Trichloroethylene (TCE): Up to 4 Orders of Magnitude above Allowed Levels [67,71].

Sample Location Identifiers	PCE (µg/L) Concentration	/0.00048 µg/L (Screening Level) = Times above Allowable Limit	To Be Removed?
NMSD3-113	8.59	1790	no
NMSD3-84	6.99	14,600	no
NMSD3-150	2.92	6100	no
NMSD3-60	2.39	5000	no
NMSD3-150/QC8-SV	1.83	3813	no
NMSD3-60/QC7-SV	1.52	3170	no
V5-5	0.811	1700	no
NMSV15-15	0.704	1500	no
V5-15	0.496	1033	no
NMSD2-150/QC6-SV	0.496	1033	no
NMSD2-150	0.384	800	no
NMSD2-130	0.288	600	no
NMSD2-92	0.091	190	no
VD1-30	0.049	102	no
NMSD2-63	0.047	98	no
NMSD2-63 DUP	0.036	75	no

17% of the 101 Ninyo and Moore [94] trichloroethylene (TCE) samples violate US Environmental Protection Agency (EPA) allowed levels, and 83% of Ninyo and Moore [94] TCE samples used tests that were 250 times too lenient to detect effects higher than allowed levels. The values above represent 100% of the samples known (through properly sensitive tests) to violate US EPA allowed TCE levels. Most illegal levels of TCE will not be removed as they are not in one of the 11 small, localized metals-hotspots [67,70], the only areas onsite to be removed.

**Table 6:** Allowed Carcinogen Exposure from Contaminated Dust, Blown Offsite ^1^ [86].

Contaminant	Allowed Contaminant Level in 50 µg/m^3^ of Dust, Blown Offsite by Wind [86]	Times above Allowed Level	Times above Allowed Level for Children Ages < 2 [5]
Carbon Tetrachloride	0.067 µg/m^3^	750	NA
Perchloroethylene	0.46 µg/m^3^	110	NA
Trichloroethylene	0.48 µg/m^3^	104	1040 ^2^

^1^ Note that, despite allowing high levels of carcinogens to be blown offsite, into surrounding neighborhoods, as the authors’ Table 3 shows, site assessors provide no required calculation of particulate-matter (PM) and other risks in ambient air. ^2^ This risk is 10 times greater for children ages 2 and younger, than for adults, given the age-dependent adjustment factor (ADAF) [5]—which applies to all onsite genotoxic carcinogens such as trichloroethylene, benzo(a)pyrene, dibenz(a,h)anthracene, methylene chloride, benz(a)anthracene, benzo(b)fluoranthene, chrysene, and indeno(1,2,3-cd)pyrene.

## Abbreviations: Acronyms Used in the Article

**Acronym**	**Phrase**
ADAF	age-dependent adjustment factor
ATSDR	US Agency for Toxic Substances Disease Registry
Cal-EPA	California Environmental Protection Agency
COPC	contaminant of potential concern
CSM	conceptual site model
CT	carbon tetrachloride
DNA	deoxyribonucleic acid
DNAPL	dense, non-aqueous phase liquid
DQA	data-quality analysis
DTSC	California Department of Toxic Substances Control
DUE	data-usability evaluation
EPA	US Environmental Protection Agency
HHSE	human health screening evaluation
IQ	intelligence quotient
MCL	maximum-allowed contaminant level
NY	New York
PAH	polyaromatic hydrocarbons
PCB	polychlorinatedbiphenyl
PCE	perchloroethylene (tetrachloroethylene)
PM	particulate matter
PPA	Prospective Purchaser Agreement
SVOC	semi-volatile organic compound
TCE	trichloroethylene
TPH	total petroleum hydrocarbons
UK	United Kingdom
US	United States
VOC	volatile organic compound
